# New Twists in Ovarian Stimulation and Their Practical Implications

**DOI:** 10.3389/fmed.2019.00197

**Published:** 2019-09-04

**Authors:** Paul Pirtea, Dominique de Ziegler, Marine Poulain, Jean Marc Ayoubi

**Affiliations:** Department of Ob Gyn and Reproductive Medicine, Hopital Foch—Faculté de Medicine Paris Ouest (UVSQ), Suresnes, France

**Keywords:** ovarian stimulation, gonadotropin, ovarian hyperstimulation syndrome (OHSS), GnRH antagonist, dual ovarian stimulation

## Abstract

Ovarian stimulation (OS) has for objective to induce multiple ovulation in order to yield a multiple oocyte harvest and offer multiple embryos available for transfer thereby increasing the efficacy of ART. Originally, the primary risk associated with OS was the occurrence of frank ovarian hyperstimulation syndrome (OHSS), a possibly dreadful—sometime fatal—complication of ART. These fears limited the number of oocytes aimed for during OS in order to curb the risk of OHSS. On the contrary, the meager implantation rates of the early days of ART led to easily transfer multiple embryos in order to achieve acceptable pregnancy rates. Today the perspectives have changed. The advent of antagonist-based OS protocol and the possibility to trigger the ultimate phase of oocyte maturation with GnRH-a has allowed to reduce the risk of OHHS. Conversely, the markedly increased implantation rates of today's ART makes multiple pregnancy a worry that has come in the limelight worldwide, pushing the practice of single embryo transfer (SET).

## Introduction

While the first successful ART pregnancy has emanated from an oocyte retrieval conducted in the natural cycle, ovarian stimulation (OS) very soon became associated to ART. Indeed, OS allows to increase the number of oocytes available and in turn the cohort of available embryos to choose from at the time of transfer. Today four decades into the history of ART, OS stands as the single most effective measure ever enacted to increase the yields—implantation and pregnancy rates—of ART. Yet the original equation that determined the constraints on the number of follicles stimulated and embryos transferred has recently changed. Indeed, the core of this brief review article focusses on the fact that a more liberal attitude generally prevails today regarding the number follicles stimulated, while efforts have focused on preferring single embryo transfers (SET).

## Oocyte Quality and Quantity

In the yesteryears of pre-ART times, the progressive decrease in fertility and increase in miscarriage rates observed as women grow older was for the larger part attributed to an aging process affecting the uterus (1). The dogma proffered that the aging uterus could not allow a proper attachment of the developing embryo. ART data have upended these views however. We now know through four decades of ART experience that the age-related decrease in implantation rates and increased in miscarriage is principally, if not solely, due to a decrease in oocyte quality. Indeed, the long recognized deterioration of the reproductive potential seen when women become older—lower pregnancy rates and increased miscarriage rates—is only seen in autologous ART. Conversely, implantation and miscarriage rates remain constant in donor-egg ART until the age of 50 years and beyond (2). This therefore clearly indicates that the decreased implantation rates and increased miscarriage rates seen in ART in older women only reflects a decrease in oocyte quality, not a uterine phenomenon (3). In aging donor-egg ART recipients, implantation, and miscarriage rates are similar to those of younger women, being solely dependent upon the age of the oocyte donor (3).

**Figure 1:**
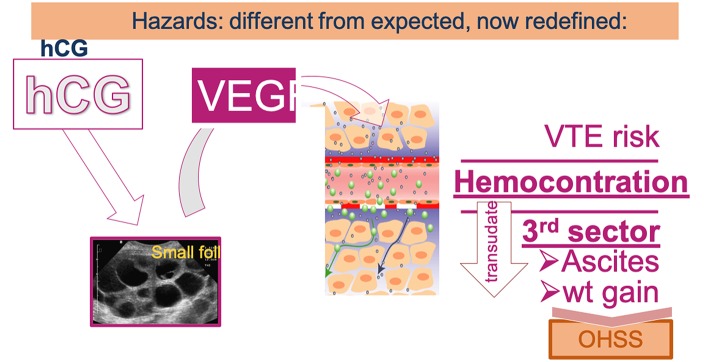
Paralell decline in oocyte quality and quantity—primordial follicle count—as a function of age (adapted from Faddy et al. with permission).

There is an erroneous belief that oocyte quality and quantity are inherently linked. Contributing to the concept that the declines in oocyte quality and quantity are linked is the fact that age induces a parallel downturn of both parameters. In an extensive study, Faddy et al. reported that the decline in total primordial follicles abruptly increases by the age of 37 years (4), as illustrated in Figure 1. The assertion that the decline in oocyte quality and quantity are fundamentally link is erroneous however. The appearance of a causal link is in fact solely the result of the effects of a confounding factor—age—which affects both oocyte quality and quantity (5). This dual effect of age explains that in older women, the so-called poor ovarian responses—insufficient oocyte yield—are commonly associated with oocytes that are also of poorer quality and lesser chances of providing a pregnancy (6). Yet as discussed below, when the decrease in oocytes is either constitutional or due to an age-independent factor, there is not necessarily a decrease in oocyte quality (7).

When oocyte quantity is impaired due to an age-independent factor such as for example endometriosis or past surgery for endometriosis, evidences indicate that oocyte quality is not decreased (7). In a retrospective cohort analysis, we showed that endometriosis is not associated with decreased oocyte quality. This was evidenced by the fact that we observed similar aneuploidy rates in endometriosis and age-matched controls (8). The fact that ART results are not necessarily reduced in endometriosis despite poorer responses to OS supports the concept that age-independent decreases in oocyte quantity are not associated with a parallel decrease in oocyte quality.

Similarly, certain women have smaller number of antral follicles despite being young. These women albeit young have poor ovarian reserve parameters such as notably, AMH levels and antral follicle count (AFC). In a series of women who had conceived and ultimately delivered a live child within 15 months of discontinuing the OC pill, we observed no correlation between AMH levels and effective time to pregnancy (9). Hence, poor ovarian reserve parameters are not *per se* associated with a decrease in natural fecundity. Likewise, a significant proportion of fertile egg donors showed a poor ovarian response to OS within a mean interval of 2 years after delivering their last child (10).

Practically therefore, a small oocyte crop obtained in women whose limited number of oocytes is not due to age warrants a different approach when managing ART than when poor responses are due to age. In the former cases, a small number of oocytes is worth harvesting because pregnancy chances are preserved contrary to what is seen in women whose poor response is due to age (6). In the latter case, a poor response— ≤ 3 oocytes expected—due to an age-related decline in ovarian response probably questions the worthiness of proceeding to oocyte retrieval.

There have been proponents of reducing the amount of gonadotropin used in OS in a strategy called mild stimulation (mOS). The purpose pursued was to reduce the number of oocytes retrieved and the possible complications of OS such as notably, OHSS while retaining a fair number of good quality oocytes. Today we know that the risk of OHSS is not due to the number of follicles responding to OS, but rather to an effect of hCG—administered for triggering ovulation or produced by the developing conceptus—on ovarian follicles. These new perspectives on OHSS certainly reduced the interest for mOS as a primary mean of curbing the risk of OHSS. Indeed, the primary defense for reducing OHSS is to avoid using hCG for triggering ovulation and proceed to a freeze all approach, which will prevent an effect trophoblastic hCG on ovarian follicles and late-onset OHSS. As this strategy has practically reduced the risk of OHSS, the justification for mild stimulation is therefore reduced. Moreover, the advent of pregestational testing for aneuploidy (PGTA) and its rapid expansion in our everyday ART practice favors increasing the number of embryos available for testing. Consonant with this view, Rodriguez-Purata and Martinez (11) states that “the aim of ovarian stimulation has shifted from obtaining embryos available for transfer to yielding the maximum embryos available for biopsy to increase the odds of achieving one euploid embryo available for transfer” (11). Nevertheless, mild OS still has its protagonists (12). These authors predict “widespread acceptance of mild IVF, by both patients and IVF providers, and make IVF more accessible to women and couples worldwide,” as mOS reduces the cost and side-effects associated with ART (12). In an editorial comment in Fertility and Sterility, Paulson R expresses similar foresights saying, “As the treatment of infertility becomes more personalized, it is likely that standard, heavy-handed stimulation protocols will give way to simpler strategies” (13). Conversely however, numerous voices also rose to indicate that mOS offers a lesser ART yield than regular OS protocols. Based on a systematic review of the literature Orvieto et al. concludes that “an objective review of the literature does not support the routine utilization of mOS in ART (14). Likewise, in a case-control study, Siristatidis et al. conclude that mOS regimens using either CC or letrozole do not seem to constitute an equally effective method as compared to the conventional OS protocols in good prognosis subfertile women (15). Further studies are therefore needed to definitively assess the role of mOS in ART. In the meantime, careful assessment of the personal history and objectives—i.e., PGTA or not—should guide clinicians in their choice of OS protocol.

## Ovarian Stimulation Does not Hamper Embryo Quality

Originally, there were concerns that excessive ovarian responses led to crops of oocytes of poorer quality (16). This diehard belief is however not supported by recent data. In one set of evidence oocyte quality was assessed by the incidence of first trimester miscarriages (17). The authors of this work based on UK registry data of 124,351 ART cases observed an inverse correlation between the number of oocytes retrieved and the risk of miscarriage (17). Of course, women who produced more oocytes tended to be younger and are therefore less prone to miscarry. Data of this study were then sub-analyzed by age groups. While as it could be expected the miscarriage risk increased with age, in each age group there was no negative impact of the number of oocytes retrieved, but rather a trend toward lesser risks of miscarriages in women who got more oocytes (17).

Concordant with the above results there was no correlation between the amounts of FSH/hMG used during OS or the number of oocytes retrieved and the euploidy risk in each age group (18). Likewise, implantation rates of euploid embryos—constant in all age groups—were not affected by the amounts of gonadotropin used during OS (18). Furthermore, the impression that there was an optimal number of oocytes retrieved−15—beyond which any further increase failed to further enhance outcome is challenged if cumulative pregnancy rates are considered. In a recent set of data, Drakopoulos et al. indicated that cumulative pregnancy rates continued to increase with oocyte retrievals that exceeded 15 oocytes (19). These authors however reported an increased risk of OHSS in the high responders. Probably however having reverted to GnRH-a only for triggering the final stages of oocyte maturation and deferred embryo transfer should have been more liberal in these cases.

These data put together with the fact that OHSS can effectively be prevented have drastically changed the terms of the OS equation. As discussed below, the current trend is to push toward harvesting more oocytes. Conversely however, the higher implantation and pregnancy rates of modern-day ART make us strive for the need of single embryo transfers (SET) whenever possible (20).

## Dual or “duplex” Ovarian Stimulation

From the onset of ART, OS was timed at the onset of the follicular phase for two primary reasons: First, to ensure that fresh embryo transfers took place during a receptive period; Second, there was an unproven belief that OS protocols had to act on antral follicles present in the early follicular phase for the fear that other hormonal environment—i.e., progesterone elevation—might negatively affect the quality of the harvested oocytes. Two factors have modified the terms of this fundamental principle that reigned over yesteryears' dogmas regarding OS in ART.

1. The advent of embryo vitrification replacing cryopreservation by the slow freezing approach freed ART from the need of transferring embryo in the luteal phase of OS without fearing a decrease in outcome. Indeed, freeze-all and deferred-embryo-transfers provide either improved or equal results as fresh transfer—based on the patient population—but never inferior results (21, 22). Hence, by freeing from the need of luteal phase transfer, OS could theoretically be initiated at times—including during the luteal phase—different than the early follicular phase (23). Reporting a meta-analysis and systematic review, Boots et al. report that luteal phase OS is equally effective as follicular phase OS even if there is a slight trend for longer stimulations and a higher dose of total gonadotropin (24).
2. The need of fertility preservation in women scheduled to initiate chemotherapy has led to initiate OS at times other than the early follicular phase—i.e., random start OS—due to the time constraints that exist in oncology. These approaches did not decrease OS efficacy (25). Further, a group in Shanghai demonstrated that luteal phase-initiated OS yielded comparable cumulative ART results—implantation and pregnancy rates—as follicular phase-started OS (23).

**Figure 2:**
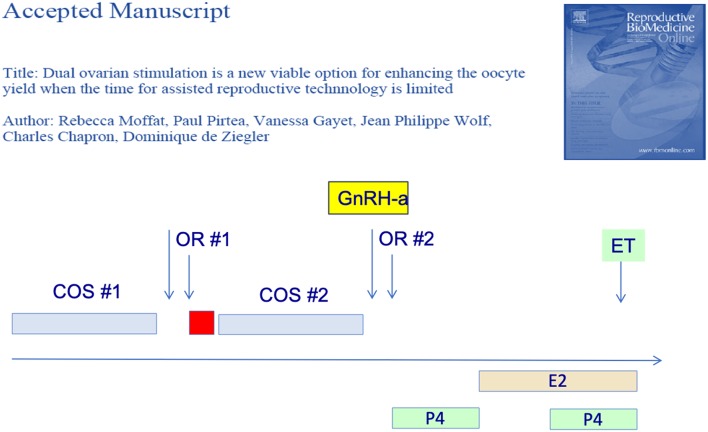
Dual or duplex ovarian stimulation during the follicular and ensuing luteal phase allow to double the number of blastocyst harvested.

Furthermore, several groups have reported that two consecutive OS protocols could be initiated in the follicular phase and the subsequent luteal phase. In the first report of this strategy—the Shanghai protocol—the authors obtained more oocytes from the luteal-phase OS. They therefore postulated that the first OS—follicular phase—exerted a priming effect on the ovarian response (26). There original protocol however used heftier OS doses during the luteal phase OS so that the priming effect of the follicular phase could not be confirmed however. Subsequently, we (27) as well as others (28) have shown that the follicular and luteal phase OS yielded a similar number of blastocysts, as illustrated in Figure 2. The latter authors (28) further showed that the embryo euploidy rates of the follicular and luteal phase were similar. The dual or “duplex” OS protocol is an effective way to increase the number of oocytes and embryo obtained over a relative short period of time. It has its place when the number of oocytes needs to be optimized over a short period of time, as for fertility preservation and in certain cases of poor responders.

Based on the duplex stimulation, the progestins (endogenous and exogenous) was administered to prevent the premature LH surge during ovarian stimulation (29) making way for new types of stimulation protocols. Two ways of using progesterone have emerged, whether endogenous, as in luteal phase stimulation, or exogenous, as in the use of progesterone in the follicular phase i.e., progestin primed ovarian stimulation (PPOS). This type of protocol in combination with the so called freeze-all strategy is also useful for OHSS-free clinics (30, 31) and also can offer a break away from the standard methodology of stimulation-retrieval-transfer.

## OHSS-free Clinics

**Figure 3:**
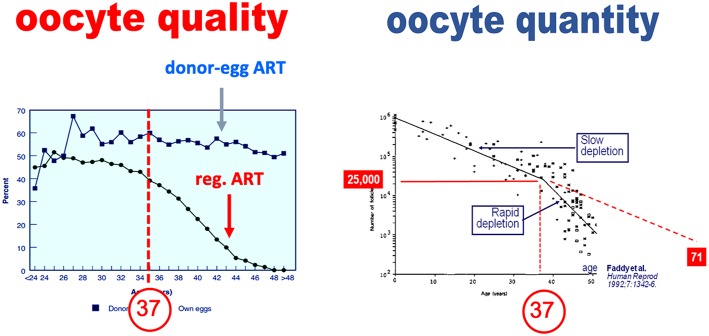
Under the influence of hCG, the hyperstimulated ovary produces the vasoactive VEGF that modifies vascular permeability leading to an efflux of vascular fluid—forming oedema and ascitis—and also leading to hemoconcentration thereby increasing the risk of venothrombo embolism (VTE).

OHSS was a dreadful complication of OS which affected up to 2–3% of ART participants in its severe form requiring hospitalization and most often ascites aspiration, which often needed to be repeated. Furthermore, OHSS was associated with an increased risk of venous thrombo-embolism (VTE) and caused several fatal outcomes. The generalization of antagonist OS protocols and the possibly to revert to GnRH-a for triggering the last step of oocyte maturation has allowed to practically eradicate the risk of OHSS (32). There are indeed evidences that OHSS stems from an effect of hCG—used for triggering ovulation or produced by the conceptus—causing the production of vasoactive endothelial growth factor (VEGF) by the developing follicles, as illustrated in Figure 3. Hence, hCG-free OS protocols as notably using GnRH-a for triggering ovulation (33)—not attempting to rescue the luteal phase with small doses of hCG—and deferred embryo transfer reduce the risk of OHSS to practically zero.

Following the use of GnRH-a for triggering ovulation the luteal phase environment is so profoundly disturbed that fresh pregnancy rates are minimal when using the common modes of luteal phase support. This led the vast majority of practitioners to revert to freeze-all-and-deferred embryo transfer each GnRH-a is used for triggering ovulation. Others have opted for different protocol of minimal hCG supplement in order to rescue the luteal phase following GNRH-a triggering of final oocyte maturation (34). Strict adherence to freeze-all-and-deferred-embryo-transfer approach when GnRH-a is used offers the valuable advantage of having reduced the most dreadful complication of ART, OHSS.

## Conclusion

Originally OS tended to be moderate for the fear of causing the dreadful—sometimes fatal—complication of ART, OHSS. Conversely, multiple embryo transfers were common to palliate the poor implantation rates that prevailed in the early days of ART and the relatively poor success of embryo freezing. Today the terms of the equation have changed. Energetic more productive and more flexible OS can be conducted without the fear of OHSS through antagonist protocols and the possibility of reverting to GnRH-a trigger for the final stage of oocyte maturation. On the contrary, the high implantation rates—notably of euploid embryos—command to most often if not always revert to SET in order eliminate the ART-increased risk in multiple pregnancy.

## Author Contributions

All authors listed have made a substantial, direct and intellectual contribution to the work, and approved it for publication.

### Conflict of Interest Statement

The authors declare that the research was conducted in the absence of any commercial or financial relationships that could be construed as a potential conflict of interest.
